# The impact of atrial fibrillation and stroke risk factors on left atrial blood flow characteristics

**DOI:** 10.1093/ehjci/jeab213

**Published:** 2021-10-23

**Authors:** Marco Spartera, Antonio Stracquadanio, Guilherme Pessoa-Amorim, Adam Von Ende, Alison Fletcher, Peter Manley, Vanessa M Ferreira, Aaron T Hess, Jemma C Hopewell, Stefan Neubauer, Rohan S Wijesurendra, Barbara Casadei

**Affiliations:** 1 Division of Cardiovascular Medicine, Radcliffe Department of Medicine, University of Oxford, Level 6, West Wing, Headley Way, Oxford OX3 9DU, UK; 2 Oxford Centre for Clinical Magnetic Resonance Research, Division of Cardiovascular Medicine, Radcliffe Department of Medicine, Oxford, UK; 3 Nuffield Department of Population Health, University of Oxford, Oxford, UK; 4 Acute Vascular Imaging Centre, Radcliffe Department of Medicine, University of Oxford, Oxford, UK

**Keywords:** left atrial myopathy, 4D flow CMR, stroke risk, atrial fibrillation, cardioversion

## Abstract

**Aims:**

Altered left atrial (LA) blood flow characteristics account for an increase in cardioembolic stroke risk in atrial fibrillation (AF). Here, we aimed to assess whether exposure to stroke risk factors is sufficient to alter LA blood flow even in the presence of sinus rhythm (SR).

**Methods and results:**

We investigated 95 individuals: 37 patients with persistent AF, who were studied before and after cardioversion [Group 1; median CHA_2_DS_2_-VASc = 2.0 (1.5–3.5)]; 35 individuals with no history of AF but similar stroke risk to Group 1 [Group 2; median CHA_2_DS_2_-VASc = 3.0 (2.0–4.0)]; and 23 low-risk individuals in SR [Group 3; median CHA_2_DS_2_-VASc = 0.0 (0.0–0.0)]. Cardiac function and LA flow characteristics were evaluated using cardiac magnetic resonance. Before cardioversion, Group 1 displayed impaired left ventricular (LV) and LA function, reduced LA flow velocities and vorticity, and a higher normalized vortex volume (all *P* < 0.001 vs. Groups 2 and 3). After restoration of SR at ≥4-week post-cardioversion, LV systolic function and LA flow parameters improved significantly (all *P* < 0.001 vs. pre-cardioversion) and were no longer different from those in Group 2. However, in the presence of SR, LA flow peak and mean velocity, and vorticity were lower in Groups 1 and 2 vs. Group 3 (all *P* < 0.01), and were associated with impaired LA emptying fraction (LAEF) and LV diastolic dysfunction.

**Conclusion:**

Patients at moderate-to-high stroke risk display altered LA flow characteristics in SR in association with an LA myopathic phenotype and LV diastolic dysfunction, regardless of a history of AF.

## Graphical Abstract

**Figure:**
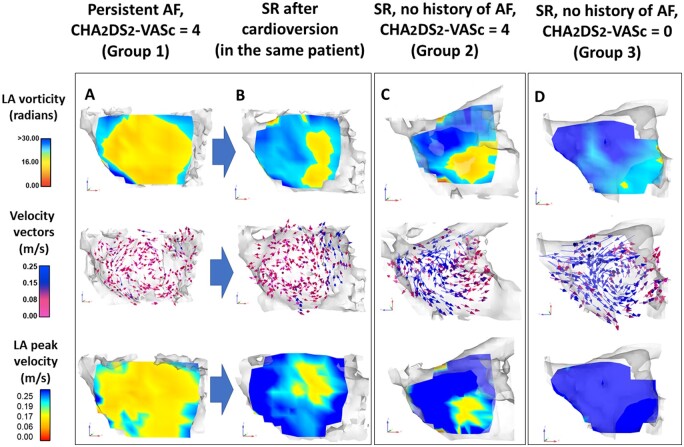

## Introduction

Atrial fibrillation (AF) is associated with profound alterations in left atrial (LA) blood flow characteristics which, in turn, increase the risk of LA thrombus formation and cardioembolic stroke.^1^ In keeping with this paradigm, anticoagulation is highly effective in preventing ischaemic stroke in patients with AF and clinical risk factors for stroke.^2^ More recently, it has been suggested that a cardioembolic aetiology may also underlie a proportion of ischaemic strokes that occur in the absence of AF, on the ground that imaging biomarkers of LA remodelling and dysfunction are associated with higher stroke risk in individuals in sinus rhythm (SR).^3^ To date, the potential mechanisms behind the association between LA myopathy and increased stroke risk remain unclear. For instance, LA remodelling could be a marker of extra-cardiac stroke risk factors, such as large-artery atherosclerosis^4^ or small vessel disease, as suggested by the association between LA dysfunction and lacunar brain infarcts.^5^ Altered LA features may also be linked to asymptomatic episodes of paroxysmal AF, with only the latter responsible for the increased stroke risk.^6^

Here, we posit that a cardiomyopathic process, brought about by exposure to cardiovascular risk factors, may lead to adverse LA blood flow characteristics (e.g. depressed LA flow velocities and vorticity) even in the absence of AF. To address this hypothesis, we assessed LA flow parameters by cardiac magnetic resonance (CMR) in patients with persistent AF, both before and after cardioversion, and in individuals in SR and with no history of AF both with or without stroke risk factors.

## Methods

This study was undertaken at the University of Oxford, UK. The study protocol was approved by a research ethics committee (18/YH/0096), and all patients gave written informed consent.

### Study design

We recruited three groups of participants: patients with persistent AF scheduled for cardioversion (Group 1); individuals in SR with no history of AF but with similar clinical stroke risk to Group 1 (Group 2); and low stroke risk individuals with no history of AF, hypertension, diabetes, heart failure, vascular disease, or stroke (Group 3). Individuals in Group 2 were offered an optional 7-day electrocardiogram (ECG) monitor to exclude the presence of asymptomatic AF episodes. The clinical stroke risk in this study was estimated using the CHA_2_DS_2_-VASc score, which estimates ischaemic stroke risk in AF^7^ and, to a lesser extent, in SR.^8^

To assess the effect of rhythm on LA flow profile, patients with persistent AF (Group 1) underwent the same CMR protocol before and at a minimum of 4 weeks after cardioversion. To assess the effect of stroke risk factors on LA flow parameters in individuals without a history of AF, patients in Group 2 were compared to low-risk individuals (i.e. Group 3).

### Recruitment of the study population

Patients with persistent AF were recruited amongst those referred for direct current cardioversion (DCCV) at the Oxford University Hospitals NHS Foundation Trust. Participants in SR were recruited from outpatient clinics, clinical CMR referrals, a pool of control participants who had participated in other research studies at our institution, or through word of mouth. Fourteen (61%) of the low-risk individuals were in-house volunteers who provided verbal consent under an ethically approved protocol.

### Cardiac magnetic resonance

**Figure 1:**
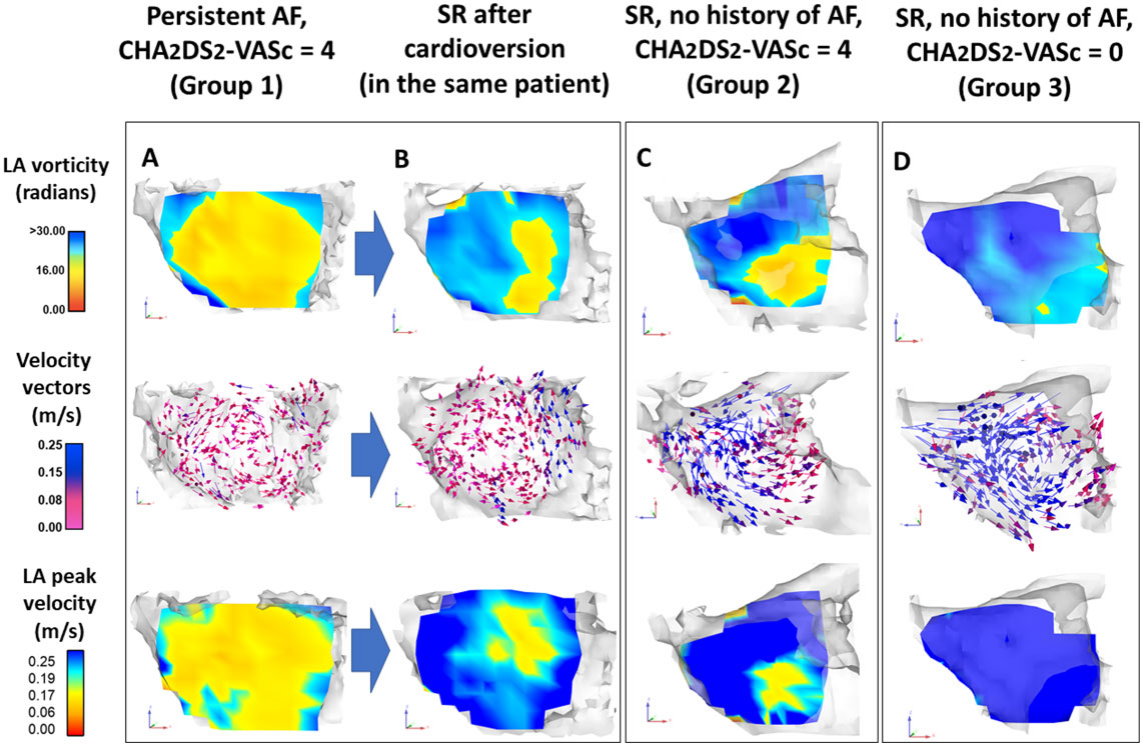
Patients at moderate-to-high stroke risk display altered LA 4D flow characteristics in SR, regardless of a history of atrial fibrillation. LA 4D flow maps in a patient with persistent AF and CHADS_2_-VA_2_Sc = 4 (Group 1, *A*) show uniformly low peak velocities (0.16 m/s) and flow vorticity (11.6 rad) compared with those obtained in the same patient forty days after successful cardioversion (peak velocity = 0.24 m/s, vorticity = 20.9 rad, *B*). Post-cardioversion 4D flow parameters in patients with a history of AF were similar to those obtained in patients with SR and no history of AF but with comparable risk factor profile (Group 2, *C*). By contrast, low-risk individuals (CHADS_2_-VA_2_Sc = 0; Group 3) in SR had a significantly higher LA peak flow velocity and vorticity compared with moderate-to-high risk patients in SR with or without a history of AF (*D*, mean data in *Figure 2*). Velocity vectors (arrows) were obtained during mid-ventricular systole. AF, atrial fibrillation; SR, sinus rhythm.

CMR examinations were performed on 3T MRI systems (Verio syngo MRB17 or MAGNETOM Prisma VE11C, both Siemens, Germany). LA flow characteristics were assessed using ECG-gated time-resolved 3D phase-contrast CMR with 3-directional velocity encoding imaging (‘4D flow’), using a post-processing tool which we have recently validated^9^ and made available on the Oxford University Research Archive platform (https://doi.org/10.5287/bodleian:ey4ovzdbB). Briefly, data analysis included 3D segmentation of LA volume, and determination of two sets of parameters (*Figure 1*): firstly, LA peak and mean velocities, since low atrial velocities are thought to be linked to activation of the coagulation cascade with increased red blood cell and plasma protein aggregation^1^; secondly, ‘rotational’ flow parameters, since the normal flow pattern within the LA is characterized by vortexes,^10^ and disruption of vortical flow is associated with altered shear stress and platelet adhesion and aggregation in computational and silicone replica models of intra-cardiac blood flow.^11^^,^^12^ Rotational parameters included mean vortex to LA volume ratio (by the lambda2 method) and LA vorticity (i.e. the tendency of flow to rotate by using the area under the vorticity–time curve over the averaged cardiac cycle using volume-weighted vorticity measures, in radians).^9^ The equations used for calculating the LA 4D flow parameters are available on the Oxford University Research Archive platform at the following link: https://doi.org/10.5287/bodleian:ey4ovzdbB. All data were acquired using a 4D flow sequence with retrospective gating except for one participant where a prospectively gated sequence was used.

The CMR protocol also included ECG-gated balanced steady-state free precession (SSFP) cine imaging in four-chamber and two-chamber orientations for evaluation of left ventricular (LV) and LA structure and function.

Sequence parameters and details of analysis methods are provided in reference^9^ and in Supplementary data online, *Methods* for LA/LV function/volumes.

All imaging assessments were conducted by investigators blinded to all clinical information, including rhythm at the time of the scan.

### Power calculation and statistical analysis

A difference of 0.06 m/s in LA peak velocity was chosen based on published data indicating that LA peak velocity in AF patients with CHA_2_DS_2_VASc ≥3 is 0.06 m/s lower than that in AF patients with CHA_2_DS_2_VASc 0–2 (13). Based on our previous work^9^ showing that LA peak velocity in patients in AF is 0.22 ± 0.07 m/s, recruitment of at least 17 participants with persistent AF would provide >90% power (two-tailed alpha = 0.05) to detect a 0.06 m/s change in peak velocity after successful DCCV. Further, recruitment of at least 22 subjects per group (i.e. SR after successful DCCV, SR participants without AF, and low-risk individuals) would provide >90% power (two-tailed alpha = 0.05 after Bonferroni correction) to detect a 0.06 m/s between-groups difference in peak velocity. Considering 35% of patients may be in AF at 4 weeks after DCCV, we aimed to recruit at least 36 patients with persistent AF.

Details of statistical tests and software used are reported in Supplementary data online, *Methods*. Briefly, normally distributed continuous variables were compared between groups with unpaired *t*-test, one-way ANOVA, or Welch’s test, whereas non-normally distributed data were compared using the Mann–Whitney *U* test or Kruskal–Wallis test. Depending on the data distribution, paired data were compared with paired *t* test or Wilcoxon signed-rank test. Categorical data were compared using the *χ*^2^ test or exact method. All tests were two-tailed, and *P-*values of <0.05 were considered statistically significant. All reported *P* values for comparisons between groups were adjusted for pairwise comparisons using the Bonferroni or Games–Howell methods.

## Results

We recruited a total of 95 participants: *N* = 37 in Group 1, *N* = 35 in Group 2, and *N* = 23 in Group 3 (*Table 1* and Supplementary data online, *Figure S1*). There were no significant differences between Group 1 and Group 2, with the exception of a lower heart rate (*P* < 0.01) and diastolic blood pressure (*P* < 0.05) in the latter. Medication use was also similar, except that anticoagulants and amiodarone were more frequently prescribed to Group 1, and anti-platelets to Group 2 (Supplementary data online, *Table S1*). Individuals in Group 3 were significantly younger and had a lower BMI than Groups 1 and 2, and also had significantly lower HR and diastolic blood pressure (DBP) than Group 1 (all *P* < 0.05). Twenty-seven patients in Group 2 (77%) agreed to undergo 7-day ECG monitoring and the remaining 8 (23%) had a resting 12-lead ECG confirming SR. Cardiac rhythm was monitored during CMR scans in all participants. No AF was found in any participant in Group 2 or 3.

**Table 1:** Baseline characteristics

	Group 1	Group 2	Group 3
	AF	SR	Low-risk individuals
*N*	37	35	23
Age, years	69 (65–75)	69 (65–73)	33 (30–57)
Male, *n* (%)	21 (57)	22 (63)	14 (61)
Body mass index, kg/m^2^	27 (25–31)	28 (25–30)	24 (22–27)
Hypertension, *n* (%)	24 (65)	26 (74)	0 (0)
Heart failure, *n* (%)	5 (13)	2 (6)	0 (0)
Stroke/TIA, *n* (%)	0 (0)	0 (0)	0 (0)
Type 2 diabetes, *n* (%)	7 (19)	10 (29)	0 (0)
Vascular disease, *n* (%)	2 (5)	7 (20)	0 (0)
CHA_2_DS_2_-VASc score	2.0 (1.5–3.5)	3.0 (2.0–4.0)	0.0 (0.0–0.0)
Heart rate, bpm	79 (74–91)	60 (55–68)	67 (60–75)
Systolic blood pressure, mmHg	123 (109–136)	121 (115–134)	114 (111–122)
Diastolic blood pressure, mmHg	77 (67–88)	65 (58–72)	67 (64–72)

Values are expressed as mean ± SD or median (Q1–Q3) for continuous variables and as *n* (%) for categorical variables. Heart rate and blood pressure were recorded at the time of the CMR scan.

TIA, transient ischaemic attack.

### CMR findings at the baseline scan

**Figure 2:**
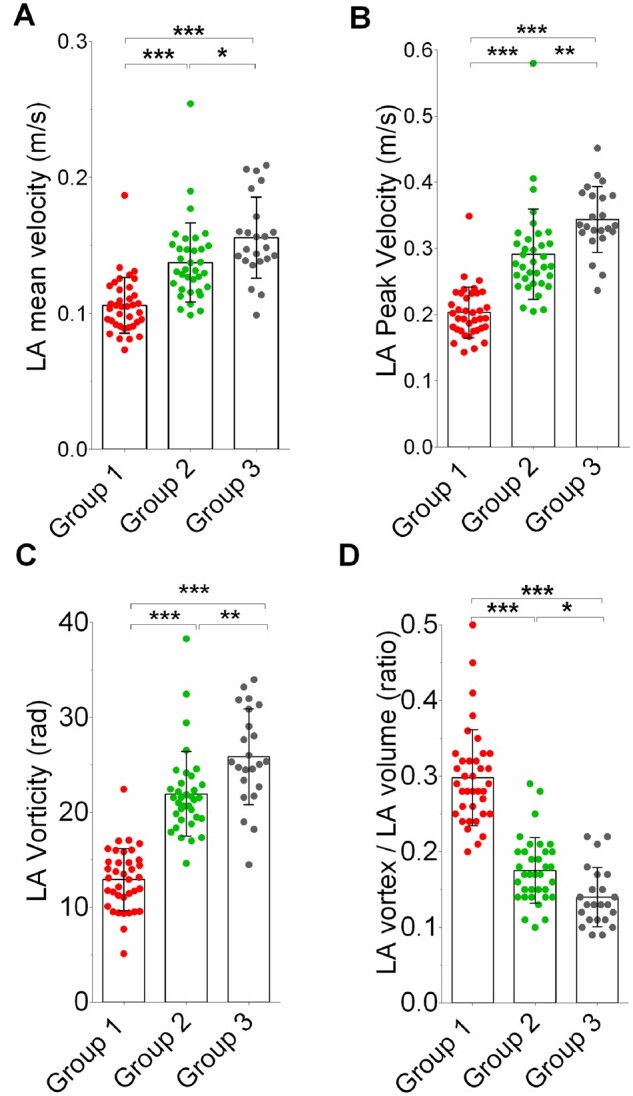
LA flow characteristics at baseline. LA mean velocity (*A*), LA peak velocity (*B*), LA vorticity (*C*), and LA vortex volume ratio (*D*) in Group 1 patients before cardioversion (in AF, in red), Group 2 patients (in SR, in green), and Group 3 low-risk individuals (in SR, in grey). Data are shown as mean ± SD. *P < 0.05; **P < 0.01; ***P < 0.001.

Patients in Group 1, all of whom were in AF at the time of the baseline scan, had significantly impaired LV systolic function (as evidenced by lower LVEF and less negative LV longitudinal strain), and adverse LA functional and structural remodelling (as evidenced by higher LA volume, lower LAEF, and impaired LA reservoir and conduit strain), compared to Groups 2 and 3 (all *P* < 0.001; Supplementary data online, *Table S2*). LA flow velocities were significantly reduced in the presence of AF compared to both groups in SR (all *P* < 0.001; *Figure 2A and B*, and Supplementary data online, *Table S2*). Further, patients with AF had significantly reduced LA flow vorticity and a greater vortex volume relative to the LA volume (all *P* < 0.001; *Figure 2C and D*, and Supplementary data online, *Table S2*).

### Effects of cardioversion on cardiac parameters and LA flow

A follow-up CMR was undertaken in all patients in Group 1 at a median of 56 days [interquartile range (IQR) 40–87 days] after the first CMR. In the intervening period, 33 patients (89%) underwent DCCV (including on two occasions in 4), 2 (6%) cardioverted pharmacologically, and 2 (6%) cardioverted spontaneously. Twenty-four patients (65%) were in SR (‘AF-SR’ subgroup) at a median of 32 days (IQR 29–41 days) after successful DCCV (*n* = 20), pharmacological cardioversion (*n* = 2, including 1 who had a previous unsuccessful DCCV), or spontaneous return to SR (*n* = 2). The remaining 13 (35%) were still in AF at the follow-up scan (‘AF-AF’ subgroup), due to primary failure of DCCV (*n* = 3), early recurrence of AF after an initially successful DCCV (*n* = 9), or early recurrence of AF after pharmacological cardioversion (*n* = 1).

There were no significant differences in patients’ characteristics between the AF-SR subgroup (from Group 1) and Group 2 (all *P* > 0.05, Supplementary data online, *Table S3*). Changes in medication between the baseline and follow-up scans were similar between the AF-AF and AF-SR groups (all *P* > 0.05, Supplementary data online, *Table S4*).

**Figure 3:**
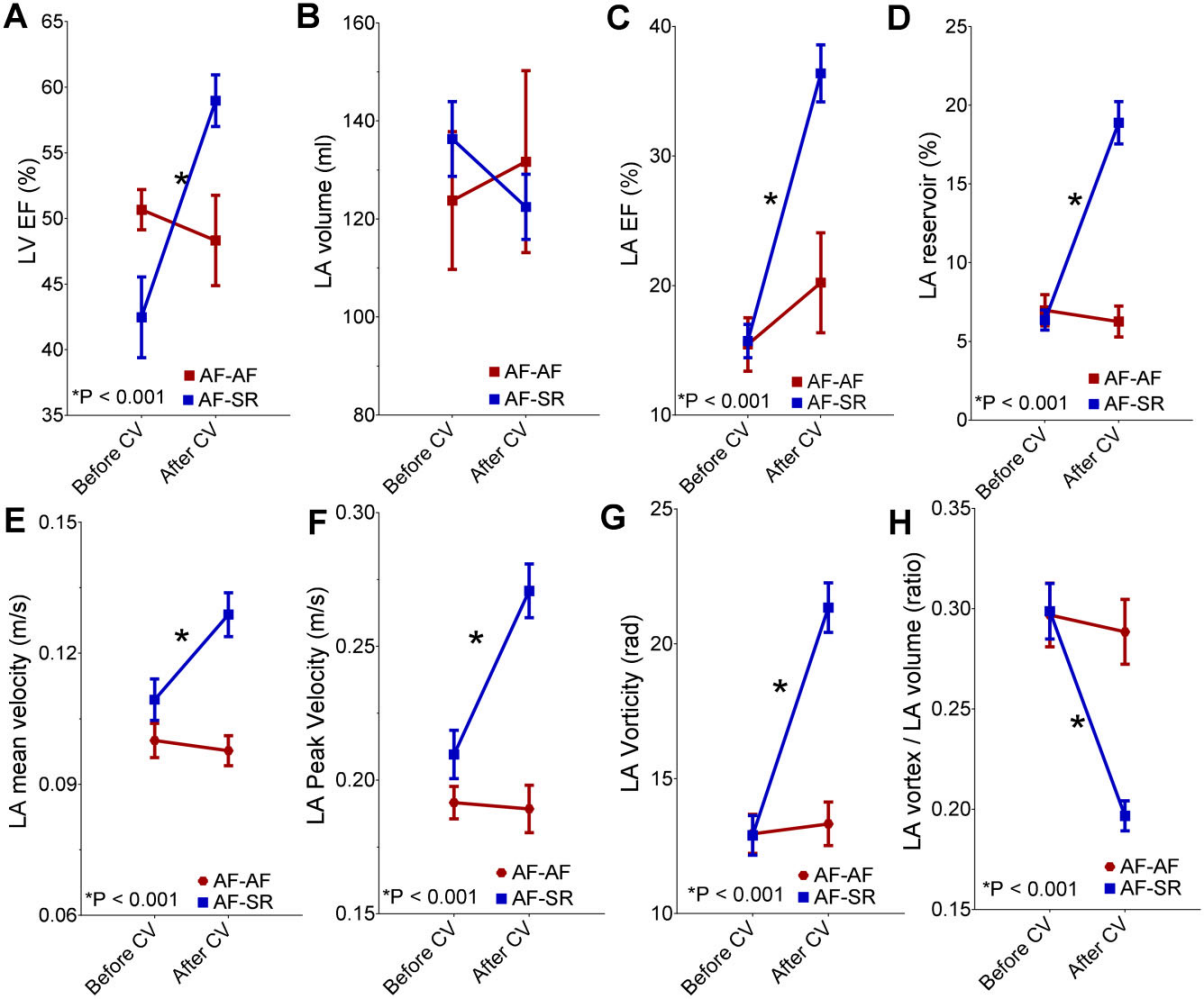
LV and LA CMR parameters in patients with persistent AF before and after cardioversion. Changes in LVEF (*A*), LA volume (*B*), LAEF (*C*), LA reservoir strain (*D*), LA mean velocity (*E*), LA peak velocity (*F*), LA vorticity (*G*), and LA vortex volume ratio (*H*) are reported in AF patients after successful restoration of SR (AF-SR subgroup, in blue) and in those with early AF relapse or failed cardioversion (AF-AF subgroup, in red). Data are presented as mean ± SE. *P < 0.05. CV, cardioversion.

Restoration of SR was associated with significant improvement in LV and LA function (all *P* < 0.001; *Figure 3* and Supplementary data online, *Table S5*). LA flow characteristics also improved, with significant increases in peak velocity, mean velocity, and vorticity, and a reduction in vortex:volume ratio (all *P* < 0.001; *Figure 3* and Supplementary data online, *Table S5*). There was no significant correlation between any LA parameter at the follow-up CMR and the time interval from restoration of SR to follow-up (all *P* > 0.05). No significant changes from baseline were observed for any parameter in patients still in AF at the follow-up CMR (*Figure 3*, all *P* > 0.05).

### Comparison between groups in SR

**Figure 4:**
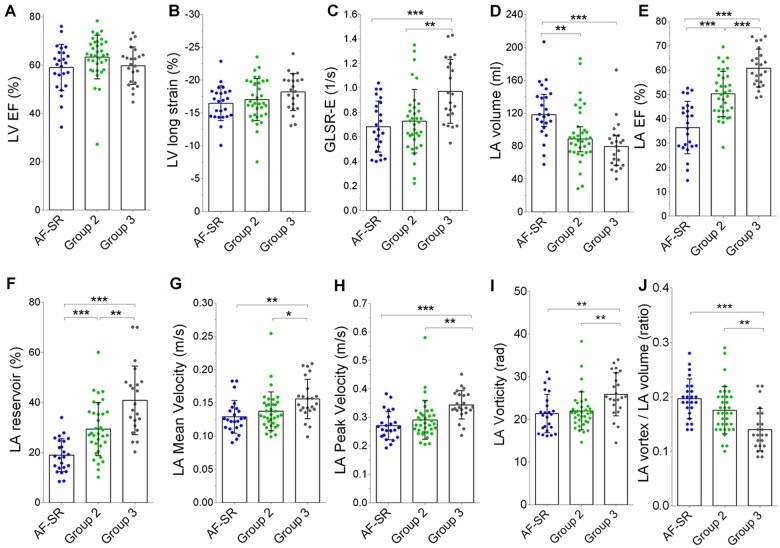
LV and LA parameters in the three groups in SR. LVEF (*A*), LV longitudinal strain (*B*), LV early diastolic strain rate (GLSR-E, *C*), LA volume (*D*), LAEF (*E*), LA reservoir strain (*F*), LA mean velocity (*G*), LA peak velocity (*H*), LA vorticity (*I*), and LA vortex volume ratio (*J*) in Group 1 patients after successful restoration of SR (AF-SR subgroup, in blue), Group 2 patients (in green) and Group 3 low-risk individuals (in grey). Data are shown as mean ± SD, with the exception of LA volume which is shown as median (IQR). *P < 0.05; **P < 0.01; ***P < 0.001.

Recovery of SR in Group 1 (i.e. in the AF-SR subgroup) abolished the difference in LV systolic function between groups (all *P* > 0.05; *Figure 4* and *Table 2*). However, both SR groups at higher clinical stroke risk (i.e. the AF-SR subgroup and Group 2) had significantly impaired LV diastolic function (as evidenced by reduced LV early diastolic strain rate) compared with the low-risk individuals in Group 3 (all *P* < 0.001; *Figure 4* and *Table 2*). Furthermore, the AF-SR subgroup continued to display LA dilatation and dysfunction compared to both groups in SR (all *P* < 0.05; *Figure 4* and *Table 2*). By contrast, the LA flow profile of AF-SR patients was now similar to that seen in SR participants in Group 2 (all *P* > 0.05), with both groups showing clear evidence of impaired LA function and flow characteristics (i.e. lower peak and mean velocity, reduced vorticity, and a higher vortex-to-LA volume ratio) compared to Group 3 (all *P* < 0.01; *Figure 4* and *Table 2*).

**Table 2:** Haemodynamics and CMR parameters in the presence of SR

	Group 1	Group 2	Group 3	*P* **value**	*P* **value**	*P* **value**
	AF-SR subgroup	SR	Low-risk individuals	1 vs. 2	1 vs. 3	2 vs. 3
N	24	35	23			
Heart rate, bpm	55 (50–64)	60 (55–68)	67 (60–75)	0.166	**0.005**	0.392
Systolic BP, mmHg	133 (113–153)	121 (115–134)	114 (111–122)	0.710	**0.013**	0.157
Diastolic BP, mmHg	73 (60–76)	65 (58–72)	67 (64–72)	0.507	0.810	>0.999
Left ventricle						
LV EDV, mL	182 ± 35	142 ± 39	170 ± 49	**0.001**	0.977	**0.041**
LVEF, %	59 ± 10	63 ± 9	60 ± 8	0.205	>0.999	0.404
LV long strain, %	−16.5 ± 2.6	−17.0 ± 3.2	−18.2 ± 2.8	>0.999	0.153	0.468
GLSR-E, 1/s	0.68 ± 0.21	0.72 ± 0.26	0.97 ± 0.26	>0.999	**<0.001**	**0.001**
Left atrium						
LA volume, mL	118 (104–143)	88 (73–102)	79 (57–93)	**0.004**	**<0.001**	0.292
LA EF, %	36 ± 11	50 ± 9	61 ± 8	**<0.001**	**<0.001**	**<0.001**
LA reservoir strain, %	18.9 ± 6.6	29.3 ± 10.6	40.8 ± 13.7	**<0.001**	**<0.001**	**0.005**
LA conduit strain, %	10.1 ± 4.3	15.3 ± 9.1	26.4 ± 11.4	**0. 015**	**<0.001**	**0.001**
LA booster strain, %	8.7 ± 3.6	14.4 ± 5.7	14.4 ± 4.9	**<0.001**	**<0.001**	0.999
LA mean velocity, m/s	0.13 ± 0.02	0.14 ± 0.03	0.16 ± 0.03	0.538	**0.002**	**0.041**
LA peak velocity, m/s	0.27 ± 0.05	0.29 ± 0.07	0.34 ± 0.05	0.579	**<0.001**	**0.003**
LA vorticity, rad	21.3 ± 4.5	22.0 ± 4.4	25.8 ± 5.0	>0.999	**0.004**	**0.007**
LA vortex/LA vol, ratio	0.20 ± 0.04	0.18 ± 0.04	0.14 ± 0.04	0.215	**<0.001**	**0.002**

Values are expressed as mean ± SD or median (Q1–Q3). Significant *P* values (<0.05 adjusted for three pairwise comparisons) are in bold.

BP, blood pressure; GLSR-E, global longitudinal strain rate—early diastolic; LAEF, LA emptying fraction; LV EDV, left ventricular end-diastolic volume; LVEF, LV ejection fraction.

**Figure 5:**
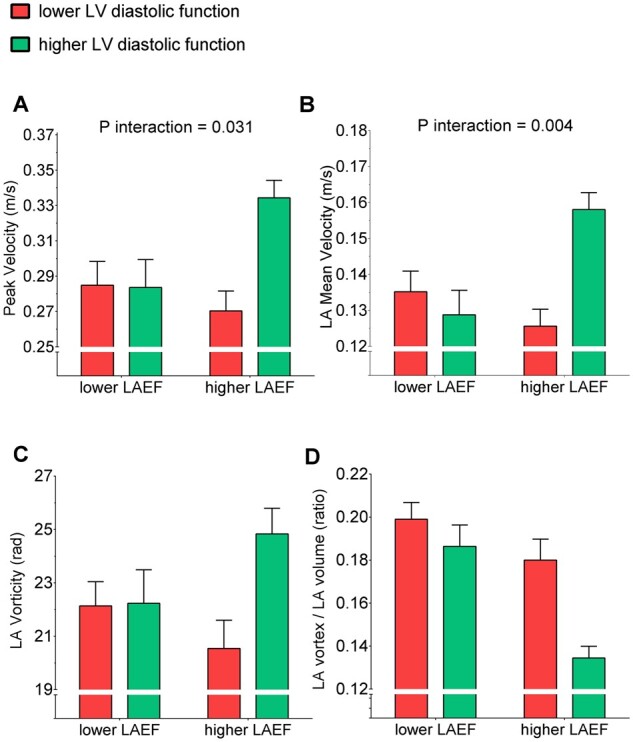
Relationships between LA emptying fraction, LV diastolic function, and LA flow Bar charts illustrate mean (SE) values of LA peak velocity (*A*), mean velocity (*B*), vorticity (*C*), and vortex/LA volume ratio (*D*), stratified by higher and lower levels of LA emptying fraction (LAEF) and LV early diastolic strain rate (GLSR-E), defined by the top 50%/bottom 50% of the distribution in SR. *P < 0.05.

When considered individually, LA and LV structural and functional parameters were weakly or non-significantly correlated with measures of LA flow (Supplementary data online, *Table S6*). However, in the sample of participants in SR (i.e. the AF-SR subgroup, Group 2, and Group 3), there was a significant interaction between LAEF and LV early diastolic function in their effects on LA peak and mean velocities (both *P* < 0.05; *Figure 5*). Specifically, whereas the most favourable LA flow profile (i.e. with higher velocities) was observed when both LAEF and LV early diastolic function were least impaired, LA flow velocities were compromised when LV diastolic function was impaired, even in the presence of preserved LAEF. After adjusting for history of AF, CHADS_2_-VA_2_Sc score, and LVEF, these interactions maintained statistical significance (all *P* < 0.05, Supplementary data online, *Table S7*).

## Discussion

We evaluated the effect of clinical stroke risk factors and cardiac function on LA blood flow characteristics in individuals in SR and in patients with persistent AF before and after cardioversion. Our results suggest that ageing and exposure to risk factors lead to a cardiomyopathic phenotype, including both LV diastolic dysfunction and reduced LA function, which is associated with lower LA flow velocities and vorticity, and a higher LA vortex volume in the presence of SR. Although altered LA flow parameters were observed in all high-risk patients in SR regardless of a history of AF, the degree of perturbation was relatively subtle when compared to the effect of AF on LA flow characteristics. These findings are in keeping with prior evidence suggesting that the AF burden is positively correlated with stroke risk,^6^ as time in AF increases the exposure to much more severely disrupted LA flow conditions than those measured in the same individual after 4 weeks from successful cardioversion. More broadly, our results suggest that at least part of the excess risk of ischaemic stroke reported in patients in SR with LA/LV dysfunction^3^ could result from the combined effect of incident AF,^14^ including asymptomatic episodes,^15^ and the underlying cardiomyopathic process^16–18^ on LA flow characteristics. This paradigm would explain the lack of a plausible temporal relationship between the majority of episodes of subclinical AF and stroke events,^19^ the relationship between AF episodes duration and incident thromboembolic events,^20^ and the results of the COMPASS trial, which showed that therapy with low-dose rivaroxaban and aspirin or rivaroxaban alone, was more effective than aspirin in reducing ischaemic stroke in patients with a high CHA_2_DS_2_-VASc score but no clinical history of AF.^21^

### LA flow characteristics and predisposition to thrombus formation

Markers of LA remodelling, such as LA size,^22^^,^^23^ LA function,^3^ and late gadolinium enhancement^24^ (as a surrogate of fibrosis), have long been associated with stroke risk in individuals in SR. This relationship may also partially reflect prediction of incident AF.^3^^,^^14^^,^^25–27^ Recent studies have reported an abnormal LA flow profile in patients with both paroxysmal and persistent AF, and showed that this is related to clinical stroke risk.^13^^,^^28^ Lower LA flow velocities are linked to activation of the coagulation cascade,^1^ whereas disruption of vortical flow is expected to alter endocardial shear stress and increase platelet adhesion and aggregation.^11^^,^^12^ This study extends these observations to patients in SR with or without a history of AF, and demonstrates that multiple flow domains are affected by both AF and stroke risk factors. Intriguingly, our results indicate that LA flow velocity only has a weak direct relationship with LA function in SR, and instead may be related to the combination of LA mechanical function and LV diastolic function, in keeping with previous epidemiological evidence of an association between LV diastolic dysfunction and ischaemic stroke in patients in SR.^29^ Overall, our results are consistent with growing evidence that ageing and long-term exposure to cardiovascular risk factors lead to a subtle atrial and ventricular cardiomyopathic phenotype,^17^^,^^30^ which, by disrupting LA flow characteristics, may increase the risk of cardioembolic events.

### Clinical implications

Measuring LA flow characteristics may improve the current assessment of thromboembolic risk in patients with or without AF. This now needs to be tested in prospective studies designed to assess the independent value of new vs. conventional LA imaging biomarkers in predicting thromboembolic events, much as the MESA cohort demonstrated with regard to the value of LAEF in predicting stroke independently of heart rhythm or LA volume.^3^ Notwithstanding their exploratory nature, the interactions reported in *Figure 5* suggest that abnormal LA flow characteristics are more likely to be present in individuals with impaired LAEF and/or LV diastolic dysfunction. If confirmed, these findings would suggest that parameters that can be derived from routine clinical imaging may be used to identify high-risk patients in SR who may benefit from anti-thrombotic therapy.

### Limitations

Patients with AF underwent follow-up CMR at 4 weeks after cardioversion. This interval was chosen as a compromise between maximizing the proportion of patients remaining in SR while also minimizing the potential confounding effects of atrial stunning. A previous study showed that stunning improves progressively after DCCV, with complete (or near-complete) resolution at 4 weeks,^31^ and current clinical guidelines recommend anticoagulation therapy for 4 weeks after DCCV in patients without a long-term indication for anticoagulation. No correlations were present between time from restoration of SR and any LA parameter at follow-up CMR, suggesting that post-cardioversion stunning was not a significant confounder in this cohort.

The relatively long 4D flow acquisition time (10–15 min) by CMR means that it is not possible to quantify beat-to-beat variations in LA haemodynamic parameters, which may be particularly accentuated in the presence of AF. Therefore, this approach is blind to possible salient flow features that may develop on an individual-beat basis.

Our 4D flow post-processing tools do not include assessment of flow within the left atrial appendage (LAA), which is the preferential site for LA thrombus formation. However, a previous study showed that in the presence of SR flow parameters in the LA and LAA are closely related.^13^ In addition, we are currently unable to assess the regionality of vortex patterns or flow at the blood/endothelium interface. Such analyses will require further technical development and are a goal for future work. Finally, the inclusion of younger (rather than age-matched) low-risk controls in Group 3 could be regarded as a limitation, although this reflected the inclusion criteria of subjects without clinical stroke risk factors, who are more likely to be younger.

## Conclusions

Compared to low-risk individuals, patients at moderate-to-high stroke risk display altered LA flow characteristics in SR, regardless of a history of AF. The abnormal LA flow profile was associated with LA dysfunction and impaired LV diastolic function.

## Supplementary data

Supplementary data are available at *European Heart Journal - Cardiovascular Imaging* online.

## Funding

The study was funded by the National Institute for Health Research (NIHR) Oxford Biomedical Research Centre and by the British Heart Foundation (BHF). V.M.F., J.C.H., and B.C. are funded by the BHF. J.C.H., A.T.H., M.S., and R.S.W. acknowledge support from the Oxford BHF Centre of Research Excellence. M.S. was funded by the NIHR Oxford BRC and acknowledges support from a competitive scholarship for young cardiologists awarded by the Italian Society of Cardiology funded by MSD ITALIA—MERCK SHARP & DOHME CORPORATION.

**Conflict of interest:** B.C. has received support in kind from Roche Diagnostics (blood assays) and iRhythm (ECG monitors) for clinical studies on AF. R.W. has received honoraria and/or travel assistance from Biosense Webster, Bayer, Boston Scientific, and Abbott. The other authors report no conflicts of interest in relation to this work.

## Data availability

The data underlying this article are available in the article and in its online supplementary material, including the link to the software we created to measure LA 4D flow.

## Supplementary Material

jeab213_Supplementary_Data
