# Supplementary material for: The impact of atrial fibrillation and stroke risk factors on left atrial blood flow characteristics
Source: Eur Heart J Cardiovasc Imaging. 2021 Oct 23;23(1):115–23. doi: 10.1093/ehjci/jeab213 (PMC8685601; doi:10.1093/ehjci/jeab213)
Supplement: jeab213_Supplementary_Data [file jeab213_supplementary_data.docx]

**SUPPLEMENTAL MATERIAL**

**Impact of Atrial Fibrillation and Stroke Risk Factors on Left Atrial Blood Flow Characteristics**

**Methods**

**Left ventricular and left atrial function**

The CMR protocol also included ECG-gated balanced steady-state free precession (SSFP) cine imaging in four-chamber and two-chamber axis orientations, for the evaluation of left ventricular ejection fraction (LVEF), and end-diastolic and end-systolic volume (LVEDV and LVESV, respectively), as well as left atrial emptying fraction (LAEF) and LA maximal and minimal volumes (LAmax and LAmin, respectively), and for derivation of LV and LA longitudinal strain and strain rate parameters by feature tracking, as described previously^1, 2^. Briefly, CMR cines were acquired with retrospective gating, including use of arrhythmia sorting as required. LAmax and LAmin were determined with the biplane area-length method and used to calculate LAEF, where: $LAEF=\frac{(LAmax -LAmin)}{\mathrm{LAmax}}$. LA volume (ml) reported in the manuscript corresponds to LAmax. LA strain values were used to assess atrial function in the 3 phases of the atrial cycle (namely, reservoir, conduit, booster) as described previously^1^. Finally, early diastolic global longitudinal strain rate (GLSR-E) was measured in sinus rhythm as the peak of the curve describing the rate (1/s) of myocardial longitudinal deformation during early diastole^2^, as a measure of LV relaxation and diastolic function^2^. LA strain values were used to assess atrial function in the 3 phases of the atrial cycle as described previously^1^ (1) LA reservoir strain (a marker of atrial distensibility) was the peak of the longitudinal strain curve during ventricular systole; (2) LA conduit strain (a marker of passive atrial conduit function during early ventricular diastole) was estimated as the difference between LA reservoir strain and LA booster strain; and (3) LA booster strain (reflecting the atrial ‘kick’ during late ventricular diastole) was the end-diastolic peak of the longitudinal strain curve.

All images were analysed using cvi42 software (v.5.3.4, Circle Cardiovascular Imaging Inc, Calgary, ON, Canada).

**Statistical analyses**

Data normality and variance were assessed by visual inspection of histograms and quantile-quantile plots, and Levene tests, respectively. Data are shown as mean ± standard deviation (SD) if normally distributed, median (Q1-Q3) if non-normally distributed, and number (percentage) if categorical, unless otherwise specified.

The unpaired Student’s t-test or one-way ANOVA were used to compare normally distributed data with homogenous variances whereas the Welch’s test was used to compare normal data with unequal variance. Non-normally distributed unpaired data were compared by using the Mann-Whitney U test or the Kruskal-Wallis test, as appropriate. Categorical data were compared by using the χ2 test, or the exact method if cell size <5. Paired data were compared with paired t test, Wilcoxon signed-rank test (for non-normally distributed variables). Correlations were calculated using Pearson’s coefficient or Spearman’s rho (for non-normally distributed variables).  The relationship between higher and lower levels of LAEF and LV function with LA flow parameters was analysed using two-way ANOVA, where groups of higher and lower levels of LAEF and LV early diastolic function were defined by the top 50% / bottom 50% of the distribution in SR.

All tests were 2-tailed, and values of P<0.05 were considered statistically significant. All reported P values for comparisons between groups have been adjusted for pairwise comparisons using the Bonferroni (for one-way ANOVA or Kruskal-Wallis or χ2 test) or Games-Howell (for Welch’s one-way ANOVA) methods.

Statistical analyses were performed with IBM SPSS Statistics for Windows, version 25.0 (IBM Corp, Armonk, NY), GraphPad Prism version 6.01 (GraphPad Software, San Diego, CA), and G*Power version 3.1.9.2.

**REFERENCES**

1. Truong VT, Palmer C, Wolking S, Sheets B, Young M, Ngo TNM, Taylor M, Nagueh SF, Zareba KM, Raman S and Mazur W. Normal left atrial strain and strain rate using cardiac magnetic resonance feature tracking in healthy volunteers. *Eur Heart J Cardiovasc Imaging*. 2019.

2. Gong IY, Ong G, Brezden-Masley C, Dhir V, Deva DP, Chan KKW, Graham JJ, Chow CM, Thavendiranathan P, Dai D, Ng MY, Barfett JJ, Connelly KA and Yan AT. Early diastolic strain rate measurements by cardiac MRI in breast cancer patients treated with trastuzumab: a longitudinal study. *Int J Cardiovasc Imaging*. 2019;35:653-662.

**Data**

**Supplementary Figure 1. Study flowchart.** We recruited 95 participants for this study including 37 individuals with persistent AF (Group 1), 35 individuals in SR with similar stroke risk to those in Group 1 (Group 2), and 23 with low stroke risk (Group 3). Every participant received at least one CMR scan. Patients in Group 1 underwent a baseline scan whilst in AF before their scheduled cardioversion, and a second scan either in SR after successful cardioversion (AF-SR subgroup) or whilst still in AF following a failed cardioversion or early relapse of AF (AF-AF subgroup). CV, cardioversion.

**Supplementary Figure 1.**


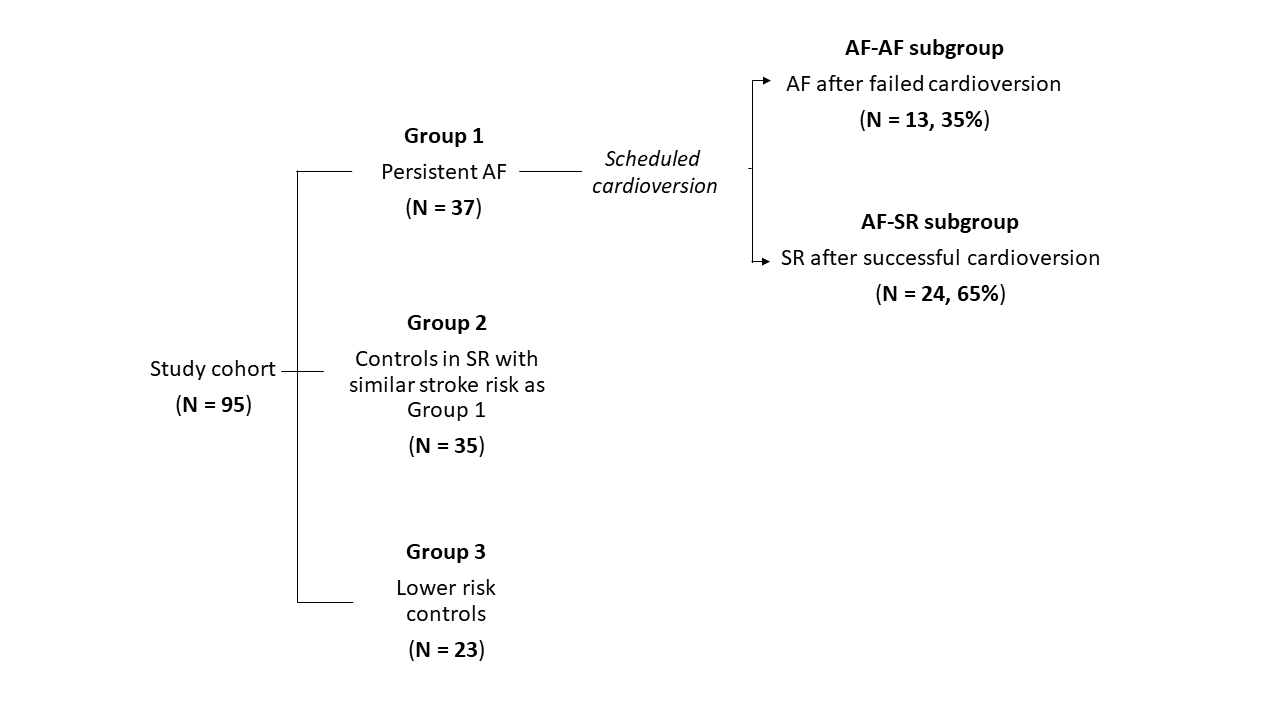


**Supplementary Table 1 – Medications at the time of baseline scan.**

| **Baseline scan** | **Group 1** | **Group 2** | **Group 3** | ***P value** |  |
| --- | --- | --- | --- | --- | --- |
| N | 37 | 35 | 23 | - |  |
| Anti-platelets | 0 (0) | 12 (34) | 0 (0) | **<0.001** |  |
| ACE inhibitor | 17 (46) | 10 (29) | 0 (0) | 0.128 |  |
| Angiotensin II receptor blocker | 5 (13) | 8 (23) | 0 (0) | 0.303 |  |
| β-blocker | 23 (62) | 18 (51) | 0 (0) | 0.358 |  |
| Aldosterone antagonist | 4 (11) | 2 (6) | 0 (0) | 0.675 |  |
| Statins | 17 (46) | 19 (54) | 0 (0) | 0.479 |  |
| Warfarin | 2 (5) | 0 (0) | 0 (0) | 0.493 |  |
| Non-vitamin K oral anticoagulants | 34 (92) | 1 (3) | 0 (0) | **<0.001** |  |
| Diuretics | 5 (13) | 9 (26) | 0 (0) | 0.191 |  |
| Calcium channel blockers | 8 (22) | 12 (34) | 0 (0) | 0.230 |  |
| Amiodarone | 10 (27) | 0 (0) | 0 (0) | **0.001** |  |
| Values are expressed as N (%). *Comparisons between patients with persistent AF (Group 1) and SR controls (Group 2). | | | | |  |

**Supplementary Table 2 – Baseline CMR characteristics**

|  | **Group 1** | **Group 2** | **Group 3** | **P value** | **P value** | **P value** |
| --- | --- | --- | --- | --- | --- | --- |
|  | **AF** | **SR controls** | **Low-risk controls** | **1 vs 2** | **1 vs 3** | **2 vs 3** |
| N | 37 | 35 | 23 |  |  |  |
| LV EDV index, ml/m^2^ | 71 ± 21 | 72 ± 19 | 89 ± 19 | $>$0.999 | **0.003** | **0.005** |
| LV EF, % | 46 ± 14 | 63 ± 9 | 60 ± 8 | **<0.001** | **<0.001** | 0.248 |
| LV longitudinal strain, % | -10.5 ± 3.7 | -17.0 ± 3.2 | -18.2 ± 2.8 | **<0.001** | **<0.001** | 0.631 |
| LA volume index, ml/m^2^ | 64 (51 - 72) | 45 (37 - 56) | 39 (32 - 49) | **<0.001** | **<0.001** | 0.510 |
| LA EF, % | 16 ± 7 | 50 ± 9 | 61 ± 8 | **<0.001** | **<0.001** | **<0.001** |
| LA reservoir strain, % | 6.6 ± 3.2 | 29.3 ± 10.6 | 40.8 ± 13.7 | **<0.001** | **<0.001** | **0.005** |
| LA conduit strain, % | 6.5 ± 3.2 | 15.2 ± 9.1 | 26.4 ± 11.4 | **<0.001** | **<0.001** | **0.001** |
| LA peak velocity, m/s | 0.20 ± 0.04 | 0.29 ± 0.07 | 0.34 ± 0.05 | **<0.001** | **<0.001** | **0.001** |
| LA mean velocity, m/s | 0.10 ± 0.02 | 0.14 ± 0.03 | 0.16 ± 0.03 | **<0.001** | **<0.001** | **0.016** |
| LA vorticity, rad | 12.9 ± 3.2 | 22.0 ± 4.4 | 25.8 ± 5.0 | **<0.001** | **<0.001** | **0.002** |
| LA vortex/LA volume ratio | 0.30 ± 0.06 | 0.18 ± 0.04 | 0.14 ± 0.04 | **<0.001** | **<0.001** | **0.022** |
| Values are expressed as mean ± SD or median (q1-q3) for continuous variables. Significant P values are shown in bold, <0.05 adjusted for 3 pairwise comparisons using Bonferroni (for one-way ANOVA or Kruskal-Wallis or Chi-square or Exact method) or Games-Howell (for Welch’s one-way ANOVA) methods. LV EDV, Left Ventricular End-Diastolic Volume; LV EF, LV Ejection Fraction; LA, Left Atrium; LAEF, LA Emptying Fraction. | | | | | | |

**Supplementary Table 3 – Clinical risk in AF patients (Group 1) who recovered SR (AF-SR) or remained in AF (AF-AF) at follow-up and in SR controls (Group 2).**

|  | **AF-AF**  **(from Group 1)** | **AF-SR**  **(from Group 1)** | **SR controls (Group 2)** | **P value (AF-SR vs Group 2)** | **P value**  **(AF-AF vs AF-SR)** |
| --- | --- | --- | --- | --- | --- |
| N | 13 | 24 | 35 |  |  |
| Age, years old | 68 (63 - 76) | 69 (65 - 74) | 69 (65 – 73) | >0.999 | >0.999 |
| Male | 4 (31) | 17 (71) | 22 (63) | >0.999 | **0.038** |
| BMI, Kg/m^2^ | 28 (24 - 30) | 27 (25 - 32) | 28 (25 - 30) | >0.999 | >0.999 |
| Hypertension | 9 (69) | 15 (62) | 26 (74) | 0.668 | >0.999 |
| Heart Failure | 2 (15) | 3 (12) | 2 (6) | >0.999 | >0.999 |
| Stroke / TIA | 0 (0) | 0 (0) | 0 (0) | - | - |
| Diabetes | 1 (8) | 6 (25) | 10 (29) | >0.999 | 0.766 |
| Vascular disease | 1 (8) | 1 (4) | 7 (20) | 0.25 | >0.999 |
| CHA₂DS₂-VASc Score | 3 (2 – 4) | 2 (1 – 3) | 3 (2 – 4) | >0.999 | 0.582 |
| Previous ablation | 3 (23) | 5 (21) | 0 (0) | - | >0.999 |
| Values are expressed as mean ± SD or median (q1-q3) or N (%). P values are adjusted for 2 pairwise comparisons using Bonferroni method. | | | | | |

**Supplementary Table 4 – CMR characteristics and medications in Group 1 patients who were successfully cardioverted at follow-up (AF-SR) versus those who remained in AF (AF-AF)**

|  | **AF-AF** | **AF-SR** | **P value** |
| --- | --- | --- | --- |
|  | N = 13 | N = 24 |  |
| HR, bpm | 79 (75 – 93) | 80 (74 - 89) | 0.962 |
| SBP, mmHg | 119 (111 - 135) | 124 (109 - 138) | >0.999 |
| DBP, mmHg | 70 (64 - 80) | 81 (69 - 92) | 0.077 |
| **CMR scan (baseline)** |  |  |  |
| LV EDV, ml/m^2^ | 56 ± 10 | 79 ± 21 | **0.001** |
| LV EF, % | 52 ± 7 | 42 ± 15 | **0.009** |
| LA Volume, ml | 106 (98 - 135) | 139 (108 - 151) | 0.109 |
| LA EF, % | 15 ± 7 | 16 ± 6 | 0.907 |
| LA reservoir strain, % | 7.0 ± 3.6 | 6.3 ± 3.1 | 0.584 |
| LA conduit strain, % | 6.9 ± 3.5 | 6.3 ± 3.1 | 0.623 |
| LA mean velocity, m/s | 0.10 ± 0.01 | 0.11 ± 0.02 | 0.157 |
| LA peak velocity, m/s | 0.19 ± 0.02 | 0.21 ± 0.04 | 0.102 |
| LA vorticity, rad | 12.9 ± 2.6 | 12.9 ± 3.6 | 0.960 |
| LA vortex/LA vol, ratio | 0.30 ± 0.06 | 0.30 ± 0.07 | 0.943 |
| Patients with changes in medications at follow-up scan | 4 (31) | 8 (33) | >0.999 |
| *Added* Amiodarone | 0 (0) | 2 (8) | 0.532 |
| *Added* Flecainide | 2 (15) | 0 (0) | 0.117 |
| *Added β-*blocker | 1 (8) | 0 (0) | 0.351 |
| *Added* statin | 0 (0) | 1 (4) | >0.999 |
| *Added* Verapamil | 1 (8) | 0 (0) | 0.351 |
| *Withheld* angiotensin-converting enzyme inhibitor | 0 (0) | 1 (4) | >0.999 |
| *Withheld β-*blocker | 0 (0) | 2 (8) | 0.532 |
| *Withheld* statin | 0 (0) | 1 (4) | >0.999 |
| *Withheld* amiodarone | 0 (0) | 1 (4) | >0.999 |
| Values are expressed as mean ± SD or median (q1-q3) or N (%). Significant P values (< 0.05) in bold. | | | |

**Supplementary Table 5 – Characteristics and CMR data in Group 1 patients who reverted to SR (AF-SR) and those who were still in AF (AF-AF) at follow-up**

|  | **AF-SR**  **(baseline)** | **AF-SR**  **(follow-up)** | **P value** | **AF-AF**  **(baseline)** | **AF-AF**  **(follow-up)** | **P value** |
| --- | --- | --- | --- | --- | --- | --- |
| N | 24 | 24 | - | 13 | 13 | - |
| Weight, Kg | 87 ± 15 | 87 ± 15 | 0.665 | 83 ± 12 | 83 ± 11 | 0.255 |
| HR, bpm | 80 (74 - 89) | 54 (50 - 64) | **<0.001** | 79 (75 - 93) | 78 (71 - 87) | 0.600 |
| SBP, mmHg | 124 (109 - 138) | 133 (113 - 153) | **0.028** | 119 (111 - 135) | 111 (107 - 126) | 0.221 |
| DBP, mmHg | 81 (69 - 92) | 73 (60 - 76) | **0.001** | 70 (64 - 80) | 72 (65 - 81) | 0.844 |
| **Left Ventricle** |  |  |  |  |  |  |
| LV EF, % | 42 ± 15 | 59 ± 10 | **<0.001** | 51 ± 5 | 48 ± 12 | 0.294 |
| LV long strain, % | -10.1 ± 4.0 | -16.5 ± 2.6 | **<0.001** | -11.2 ± 3.2 | -13.1 ± 2.9 | 0.094 |
| **Left Atrium** |  |  |  |  |  |  |
| LA Volume, ml | 139 (108 - 151) | 118 (104 - 143) | 0.067 | 106 (98 - 135) | 121 (93 - 145) | 0.422 |
| LA EF, % | 16 ± 6 | 36 ± 11 | **<0.001** | 15 ± 7 | 20 ± 14 | 0.281 |
| LA reservoir strain, % | 6.3 ± 3.1 | 18.9 ± 6.6 | **<0.001** | 7.0 ± 3.6 | 6.2 ± 3.5 | 0.154 |
| LA conduit strain, % | 6.3 ± 3.1 | 10.1 ± 4.3 | **<0.001** | 6.9 ± 3.5 | 6.2 ± 3.5 | 0.212 |
| LA mean velocity, m/s | 0.11 ± 0.02 | 0.13 ± 0.02 | **<0.001** | 0.10 ± 0.01 | 0.10 ± 0.01 | 0.733 |
| LA peak velocity, m/s | 0.21 ± 0.04 | 0.27 ± 0.05 | **<0.001** | 0.19 ± 0.02 | 0.19 ± 0.03 | 0.971 |
| LA vorticity, rad | 12.9 ± 3.6 | 21.3 ± 4.5 | **<0.001** | 12.9 ± 2.6 | 13.3 ± 2.9 | 0.429 |
| LA vortex/LA vol, ratio | 0.30 ± 0.07 | 0.20 ± 0.04 | **<0.001** | 0.30 ± 0.06 | 0.29 ± 0.06 | 0.693 |
| Values are expressed as mean (SD) or median (q1-q3). Significant P values (<0.05) for paired comparisons in bold. LV EDV, Left Ventricular End-Diastolic Volume; LV EF, LV Ejection Fraction; LV early diastolic Strain Rate, GLSR-E; LA Emptying Fraction, LAEF. | | | | | | |

**Supplementary** **Table 6 – Correlation coefficients between LA flow and structural/functional parameters in the whole population in SR at the time of the scan.**

| **N=82** | LVEF |  |  |  |  |  |  |  |  |  |
| --- | --- | --- | --- | --- | --- | --- | --- | --- | --- | --- |
| LV long strain | **-0.634^**^** | LV long strain |  |  |  |  |  |  |  |  |
| LV early diastolic strain rate | 0.233^*^ | **-0.546^**^** | LV early diastolic strain rate |  |  |  |  |  |  |  |
| LA volume | -0.092 | 0.099 | -0.104 | LA volume |  |  |  |  |  |  |
| LAEF | 0.248^*^ | -0.426^**^ | 0.333^**^ | **-0.577^**^** | LAEF |  |  |  |  |  |
| LA reservoir strain | 0.305^**^ | **-0.514^**^** | 0.441^**^ | **-0.512^**^** | **0.813^**^** | LA reservoir strain |  |  |  |  |
| LA conduit strain | 0.217 | -0.457^**^ | **0.584^**^** | -0.429^**^ | **0.695^**^** | **0.913^**^** | LA conduit strain |  |  |  |
| LA mean velocity | 0.227^*^ | -0.194 | 0.329^**^ | -0.317^**^ | 0.336^**^ | 0.382^**^ | 0.366^**^ | LA mean velocity |  |  |
| LA peak velocity | 0.181 | -0.208 | 0.328^**^ | -0.334^**^ | 0.358^**^ | 0.391^**^ | 0.375^**^ | **0.938^**^** | LA peak velocity |  |
| LA vorticity | 0.121 | -0.168 | 0.302^**^ | -0.092 | 0.260^*^ | 0.261^*^ | 0.296^**^ | **0.611^**^** | **0.689^**^** | LA vorticity |
| LA vortex volume ratio | -0.084 | 0.373^**^ | -0.456^**^ | 0.205 | **-0.599^**^** | -0.506^**^ | -0.453^**^ | -0.383^**^ | -0.371^**^ | -0.338^**^ |
| Pearson’s coefficient and Spearman’s rho are reported as appropriate in the study population in sinus rhythm at the time of the scan (namely, successfully cardioverted AF patients in Group 1, matched SR controls in Group 2, and healthy controls in Group 3). Table entries with * indicate statistically significant relationships (2-tailed P < 0.05) and smaller P values stratified by size (**P < 0.01). Moderate correlation (coefficient > 0.5) is marked in bold. Strong correlation (coefficient equal or greater than 0.7) is marked in bold with light grey background | | | | | | | | | | |

**Supplementary Table 7 – Relationships between LA emptying fraction, LV diastolic function, and LA flow after adjustment for covariates in SR groups**

| **Independent variables** | **Dependent variable** | **Adjustment Covariates** | **P value** |
| --- | --- | --- | --- |
| GLSR-E low/high levels | LA peak velocity | - | **0.005** |
| LAEF low/high levels | LA peak velocity | - | **0.029** |
| GLSR-E * LAEF low/high levels | LA peak velocity | - | **0.031** |
| GLSR-E * LAEF low/high levels | LA peak velocity | LVEF | **0.025** |
| GLSR-E * LAEF low/high levels | LA peak velocity | CHA_2_DS_2_VASc | **0.034** |
| GLSR-E * LAEF low/high levels | LA peak velocity | History of AF | **0.047** |
| GLSR-E * LAEF low/high levels | LA peak velocity | Age | 0.085 |
| GLSR-E * LAEF low/high levels | LA peak velocity | CHA_2_DS_2_VASc, History of AF, Age | 0.118 |
| GLSR-E low/high levels | LA mean velocity | - | **0.006** |
| LAEF low/high levels | LA mean velocity | - | **0.018** |
| GLSR-E * LAEF low/high levels | LA mean velocity | - | **0.004** |
| GLSR-E * LAEF low/high levels | LA mean velocity | LVEF | **0.002** |
| GLSR-E * LAEF low/high levels | LA mean velocity | CHA_2_DS_2_VASc | **0.004** |
| GLSR-E * LAEF low/high levels | LA mean velocity | History of AF | **0.006** |
| GLSR-E * LAEF low/high levels | LA mean velocity | Age | **0.011** |
| GLSR-E * LAEF low/high levels | LA mean velocity | CHA_2_DS_2_VASc, History of AF, Age | **0.018** |
| General linear regression models in the study population in sinus rhythm at the time of the scan (namely, successfully cardioverted AF patients in Group 1, matched SR controls in Group 2, and healthy controls in Group 3). Statistically significant P values (<0.05) are marked in bold. LAEF, LA emptying fraction. LVEF, LV ejection fraction. GLSR-E, LV early diastolic strain rate. * expresses interaction terms | | | |
